# Routing Protocols for Underwater Wireless Sensor Networks: Taxonomy, Research Challenges, Routing Strategies and Future Directions

**DOI:** 10.3390/s18051619

**Published:** 2018-05-18

**Authors:** Anwar Khan, Ihsan Ali, Abdullah Ghani, Nawsher Khan, Mohammed Alsaqer, Atiq Ur Rahman, Hasan Mahmood

**Affiliations:** 1 Department of Electronics, University of Peshawar, Peshawar KPK 25000, Pakistan; 2 Department of Electronics, Quaid-i-Azam University, Islamabad 44000, Pakistan; hasan@qau.edu.pk; 3 Department of Computer System and Technology, Faculty of Computer Science and Information Technology, University of Malaya, Kualalumpur 50603, Malaysia; 4 School of Computing and IT, Taylor’s University, Kualalumpur 50603, Malaysia; 5 Collage of Computer Science, King Khalid University, Abha 61421, Saudi Arabia; nawsherkhan@gmail.com; 6 Department of Computer Science, Abdul Wali Khan University, Mardan 23200, Pakistan; msalsaqr@kku.edu.sa; 7 Faculty of Computing and Information Technology, Northern Border University, Rafha 11351, Saudi Arabia; atiq621@gmail.com

**Keywords:** survey, routing, underwater wireless sensor networks (UWSNs), protocol, localization

## Abstract

Recent research in underwater wireless sensor networks (UWSNs) has gained the attention of researchers in academia and industry for a number of applications. They include disaster and earthquake prediction, water quality and environment monitoring, leakage and mine detection, military surveillance and underwater navigation. However, the aquatic medium is associated with a number of limitations and challenges: long multipath delay, high interference and noise, harsh environment, low bandwidth and limited battery life of the sensor nodes. These challenges demand research techniques and strategies to be overcome in an efficient and effective fashion. The design of routing protocols for UWSNs is one of the promising solutions to cope with these challenges. This paper presents a survey of the routing protocols for UWSNs. For the ease of description, the addressed routing protocols are classified into two groups: localization-based and localization-free protocols. These groups are further subdivided according to the problems they address or the major parameters they consider during routing. Unlike the existing surveys, this survey considers only the latest and state-of-the-art routing protocols. In addition, every protocol is described in terms of its routing strategy and the problem it addresses and solves. The merit(s) of each protocol is (are) highlighted along with the cost. A description of the protocols in this fashion has a number of advantages for researchers, as compared to the existing surveys. Firstly, the description of the routing strategy of each protocol makes its routing operation easily understandable. Secondly, the demerit(s) of a protocol provides (provide) insight into overcoming its flaw(s) in future investigation. This, in turn, leads to the foundation of new protocols that are more intelligent, robust and efficient with respect to the desired parameters. Thirdly, a protocol can be selected for the appropriate application based on its described merit(s). Finally, open challenges and research directions are presented for future investigation.

## 1. Introduction

The planet Earth consists of 70% water. This shows the importance of exploring the underwater medium. The sensor nodes in UWSNs cover a specific area of the sea to sense some attributes and inform the onshore data center near the water surface [1]. In forwarding data packets from the bottom to the surface of the water, the sensor nodes communicate with one another to identify and use the paths that are the best with respect to some selection criterion(a). Routing protocols for UWSNs deal with the selection of such paths to deliver data packets to the surface destination in an efficient and effective fashion. Recently, researchers, scientists and engineers have used the routing protocols to investigate the underwater medium for a number of applications. They include monitoring of the underwater environment for military and civilian purposes, forecasting disasters, leak detection [2] and general underwater exploration [3].

The underwater medium is highly challenging and unpredictable due to a number of reasons [4]. Water has the property of absorbing radio frequency (RF) waves. This increases the attenuation and causes energy loss of RF waves in water. For a specific frequency *f* in Hz (Hertz), the rate at which water absorbs the energy of the radio waves is $45\sqrt{f}$ dB/km (decibels per kilometer). Water becomes a conductor of the RF waves at extra low frequencies in the range of 30–300 Hz. However, this frequency range requires a very large antenna size for the transmitter with a high transmit power. This requirement is impractical and, consequently, the RF waves are not used in underwater communications. The use of the optical waves is also not effective in the underwater environment. This is because the optical waves require high precision to point them between a transmitter and a receiver, while the underwater medium is unpredictable and sensor nodes move with the water currents. In essence, acoustic waves are used in UWSNs. However, the speed of acoustic waves is almost five-times in magnitude slower than the speed of RF waves. This causes high propagation delay in underwater communications.

The use of acoustic waves limits the range of useful frequencies in underwater communications as the acoustic spectrum is limited [5]. Furthermore, the speed of an acoustic wave changes significantly with depth, salinity and temperature of the water. This causes the acoustic waves to travel on curved paths in water, which as a result, creates zones where sensor nodes are inaudible. Such sensor nodes then do not participate in the data transmission process, which affects the performance of the network. The marine life, shadow zones and unpredictable nature of the underwater medium with high interference and severe noise further threaten the reliable delivery of data packets from the bottom to the surface of the water. Moreover, the sensor nodes have limited battery life, and it is inefficient to replace the batteries of the sensor nodes [6], especially at the ocean’s bed.

The design of routing protocols for UWSNs is of paramount importance. These protocols identify paths from the bottom to the surface of the water to ensure network performance in accordance with the desired parameters. Specifically, the challenges associated with the underwater medium and during packet forwarding are considered by these protocols to achieve the optimal performance of the network according to the desired goals. For instance, these protocols cope with the limited battery power, severe noise and interference, shadow zones, movements of the sensor nodes with water currents, reliable delivery of data packets during unfavorable channel conditions and high propagation delay [7].

There are many surveys conducted on routing protocols for UWSNs in the literature [8,9,10,11]. However, these surveys are either not too focused on routing protocols or do not consider the recent routing protocols for UWSNs. In addition, some of the surveys do not address the parameters such as routing strategies of the addressed protocols or their merits and demerits. A description of these parameters is essential for the researchers, engineers and scientists that design and test the routing algorithms for UWSNs. The description of these parameters helps in the selection of the right protocol for the right application in UWSNs. Moreover, these parameters also help researchers to design new routing strategies based on the addressed flaws in the addressed routing schemes. This, in turn, leads to the foundation of new routing protocols that are more robust, intelligent, efficient and smart as compared to the addressed protocols.

In this paper, a survey of the routing protocols for UWSNs is given. As compared to the existing surveys, this survey considers only recent and state-of-the-art protocols (designed in the last one or two years except some pioneering protocols). The protocols are classified into two categories: localization-based and localization-free protocols. The former type requires the two- or three-dimensional position coordinates information of the sensor nodes, while the latter type requires only the pressure of the water on the sensor nodes (depth) to identify the routing trajectories. Further division of the two categories of protocols is into multiple subcategories based on the problems they address or the major parameters they consider during routing. The subdivision includes categories based on the nodes’ mobility, energy balancing, channel properties, energy consumption and the void zone. This division and subdivision are helpful and effective for researchers to understand the theme of the design of every protocol. Unlike the existing surveys, the survey particularly focuses on the routing strategies, merits and demerits of the addressed protocols. The description of the routing strategies helps researchers understand the routing process of the addressed protocols. The description of the merit(s) of each protocol eases its selection by researchers for a particular application, depending on the nature of the application. For instance, in time-sensitive applications, such as military surveillance and disaster prevention, a protocol selecting the shortest path to shorten the propagation delay is the most effective as compared to other protocols. In a similar fashion, applications such as underwater pollution detection and equipment monitoring (where time efficiency is not desired) require that the network operates for a significantly long time. Therefore, the selection of energy-efficient routing protocols suffices for the requirements of these applications.

Describing the demerits of the protocols is useful in the design of new routing protocols for UWSNs that are more effective. This is because new protocols can be designed keeping in view the demerits of the existing protocols. In this way, more robust, efficient and smarter protocols can be designed. Moreover, the challenges associated with the underwater medium are addressed, and the protocols are described in terms of addressing these challenges, as well as the challenges that arise during packet forwarding. Finally, open research challenges and future research directions are highlighted for the researchers, scientists and engineers.

In essence, the following are the contributions of this paper:

- The current and state-of-the-art routing protocols for UWSNs that are mostly not addressed in the existing surveys are considered. Unlike the existing surveys, every protocol is described in terms of its routing strategy, merit(s), demerit(s) and latency. The merit(s) of every protocol is (are) linked with the cost. For instance, the high delivery of packets to the final destination of some protocols is linked with their high energy consumption. This, in turn, leads to a short network lifetime. The routing strategy of every protocol is described to help researchers understand the protocol conveniently. The description of the merit(s) of each protocol makes the selection of the right protocol by researchers, scientists and engineers convenient for the right application. The description of the demerit(s) of every protocol, on the other hand, leads to the foundation for the design of new and novel protocols free of the addressed demerit(s). The latency information helps the researcher to select novel protocols.
- The addressed routing protocols are classified into two categories: localization-based and localization-free protocols. The former class of protocols requires the two- or three-dimensional position coordinates information of the sensor nodes to find routing trajectories. The latter class requires only the depth or pressure of the sensor nodes to identify routing paths. Each category of the protocols is further classified based on the problem(s) it addresses or the major parameter(s) it considers during routing. The parameters for subdivision include nodes’ mobility, energy balancing, channel properties, energy consumption and void zone. This classification helps the researchers in the selection of the appropriate protocols for the desired applications. For instance, in applications where scalability is required, such as underwater exploration, a localization-free routing protocol is a better choice than a localization-based routing protocol.
- Challenges associated with the underwater medium and during packet forwarding from the bottom to the surface of water are described. The operation of every protocol is linked with how these challenges are addressed and mitigated.
- Open research challenges and future research directions are specified based on the described protocols, existing issues and challenges.

## 2. Challenges in Underwater Communications

Routing protocols are designed for UWSNs keeping in view the challenges in underwater communications. This necessitates the description of these challenges before the routing protocols are described in detail. These challenges are highlighted in the lines to follow.

### 2.1. Underwater Noise

In general, underwater noise adds to the desired data and degrades the quality of communications. As a remedy, routing protocols have to select the paths less affected by noise. The underwater ambient noise is constituted by four components: shipping, wave, thermal and turbulence noise [12]. The power spectral density *N* of the ambient noise in dB is the sum of the power spectral densities of the four noise types and is given by:

(1) $$N=N_{sh}+N_{wv}+N_{tb}+N_{th}$$

where $N_{sh}$, $N_{wv}$, $N_{tb}$ and $N_{th}$ are the power spectral densities of the shipping, wave, turbulence and thermal noise, respectively. They are modeled by:

(2) $$N_{sh}=40+20\left(s-0.5\right)+26\log\left(f\right)-60\log\left(f+0.03\right)$$

(3) $$N_{wv}=50+7.5^{w}+20\log f-40\log\left(f+0.4\right)$$

(4) $$N_{tb}=27-30\log f$$

(5) $$N_{th}=-27+\log f$$

where *f* is the frequency in kHz, *w* is the speed of wind in m/s and s∈[0,1] determines the extent of shipping activities in water. Turbulence in water generates noise that corrupts frequencies below 20 Hz. The noise generated by shipping activities ranges from 20–200 Hz. Waves generated by wind blowing at the surface of water with speed *w* result in noise generation from 200–200 kHz. Temperature-generated thermal noise affects acoustic frequencies above 200 kHz.

### 2.2. Channel Attenuation

The attenuation in underwater communications is the result of absorption loss and spreading loss [12]. This, in effect, decreases the strength of the desired signals as the routing process carries on. As a result, it becomes difficult to extract the desired data from the received signal at the final destination. The attenuation in dB of an acoustic wave of frequency *f* in kHz at a distance *d* from the source is denoted by A(d,f) and is modeled by:

(6) $$A\left(d,f\right)=A_{o}d^{k}\alpha {\left(f\right)}^{d}$$

where $A_{o}$ is the normalization constant, α is the absorption coefficient and *k* is the spreading factor. The geometry in which an acoustic wave spreads away from the source determines the value of *k*. Cylindrical and spherical spreading geometries are represented by k=1 and k=2, respectively. In practice, k=1.5 [12].

### 2.3. Limited Bandwidth

The harsh underwater medium allows only specific frequencies in the acoustic spectrum to carry information [12]. This restricts the available bandwidth, which in turn, puts restrictions on the design of the acoustic systems. Consequently, routing protocols have to take into account the limited range of available frequencies to select the routing paths and deliver data packets to the final destination. The convergence (transmission range) of an underwater application is inversely proportional to its bandwidth, as shown in Table 1 [1].

### 2.4. The Speed of Acoustic Waves

The speed of an acoustic wave used in underwater communications varies with respect to salinity, temperature and depth of the water. The speed of acoustic waves in water is modeled by [13]:

(7) $$\begin{array}{ccc} c & = & 1449+4.591T-5.304\times 10^{-2}T^{2}\\ & & +2.374\times 10^{-4}T^{3}+1.34\left(S-35\right)\\ & & +1.63\times 10^{-2}D+1.675\times 10^{-7}D\\ & & +1.025\times 10^{-2}T\left(S-35\right)-7.139\times 10^{-3}TD^{3} \end{array}$$

where the parameters *c*, *T*, *S* and *D* represent the speed of acoustic waves in m/s, temperature in ${}^{\circ }$C (degrees Celsius), salinity factor in ppt (parts per thousand) and the depth of sea in meters, respectively. The above equation holds under the conditions $0{\;}^{\circ }$C $<T\leq 30{\;}^{\circ }$C, 0 m ≤D≤8000 m and salinity in the range 30–40 ppt. The slower speed of acoustic waves than RF waves brings an inherently higher propagation delay in underwater communications than the terrestrial RF communications.

Variations in the speed of an acoustic wave affect the delivery time of data during routing. This specifically challenges the design of routing protocols for time-critical underwater applications such as disaster prediction and prevention, military surveillance and rescue operations.

### 2.5. Short Network Lifetime

The limited battery power of the sensor nodes in UWSNs results in a short network lifetime [14]. As described before, battery replacement is not an efficient solution. As soon as the sensor nodes start depleting their battery power, energy holes (dead nodes) start to be created. This, in effect, starts the degradation of the network performance and hinders the delivery of data packets to the surface of the water. This limitation demands for efficient and effective use of the electrical power of sensor nodes in UWSNs.

Routing protocols for UWSNs need to optimally select the routing paths with respect to the energy consumption. This prolongs the network lifetime. If nodes drain their battery power at an early stage of network operation, they become dead and are called energy holes. Such energy holes then interrupt packet delivery to the destination.

## 3. Underwater Routing Protocols

In this section, all the addressed routing protocols for UWSNs are categorized into two types: localization-based and localization-free protocols. Each type is further classified according to the problem(s) it addresses or the major parameter(s) it utilizes for routing purpose. These protocols are further described in the lines to follow.

### 3.1. Localization-Based Routing Protocols

These protocols require that the two- or three-dimensional coordinates information of the sensor nodes be known for determining the routes from the bottom to the surface of the water. Without the coordinates information, the distances among nodes and the routing trajectories cannot be determined in these protocols. These protocols are further classified into the following types based on the problems they address, resolve or the parameters they utilize to make the routing decisions. Table 2 shows a summary of these protocols.

#### 3.1.1. Protocols Addressing Nodes’ Mobility

These protocols make use of the mobility of the sensor nodes in data routing. The mobility of the sensor nodes is either used as a parameter to be controlled or to help other nodes to select different forwarder nodes during data forwarding. These protocols are described in the lines to follow.

##### VBF

Vector-based forwarding (VBF) addresses the mobility issue of the underwater sensor nodes [15]. It predefines a virtual pipe from a source to a destination. Only nodes that are located within the pipe qualify for data forwarding. Every packet contains information about the position of the source, forwarder and destination. When a node receives a packet, it calculates its position. If the calculated position is such that it lies within the pipe, the position information is inserted by the node in the packet, and it sends the packet to the next forwarder. Otherwise, the packet is discarded. Since the protocol limits the number of forwarders in data transmission, it achieves energy efficiency. Furthermore, it is not necessary to know the state information of each node that makes the network easily scalable. However, nodes within the pipe die soon due to the frequent selection for data forwarding. The death of these nodes creates energy holes and, in turn, results in packet loss. In addition, there may be no forwarder at all within the pipe when the network is sparse.

##### SBR-DLP

The sector-based routing with destination location prediction (SBR-DLP) protocol makes the destination node mobile rather than fixed at the surface of the water [16]. This makes all nodes know only their position information and predict the predefined mobility pattern of the destination node. A node does not need to know the position information of its neighbors. A source node that has data to send broadcasts a control message to its one-hop neighbors that contains its position information and packet ID. Upon receiving the control packet, every neighbor node replies to the source node only if it is closer to the destination node (the predefined position of the destination) than the source node. The packet collision is overcome by dividing the transmission range of the source node into sectors. Nodes in the sector bisected by the straight line from the source to the destination are prioritized to send replies to the source node. The protocol achieves a significant reception of packets at the sink when nodes are mobile. However, it has high delay because the mobile nodes do not prioritize the nodes that have data packets ready for transmission. Instead, the mobile nodes move with their predefined paths irrespective of which node has data packets ready for transmission.

##### NEFP

The novel efficient forwarding protocol (NEFP) performs three tasks: defines the routing zone to avoid unnecessary forwarding, calculates holding time to avoid packet collision and uses Markov chains to estimate the forwarding probability of packets in the varying topology [17]. However, the performance of the proposed protocol degrades in sparse conditions when the probability of forwarding a packet fairly reduces as forwarders are less likely to find in the defined forwarding zones.

##### TC-VBF

The topology control VBF (TC-VBF) addresses the issue of nodes’ communication in sparse conditions and selects forwarders in the manner of VBF in addition to considering the density of the network [18]. However, it suffers from the same problems and issues as VBF, described above.

##### LBDR

The authors in [19] proposed a localization-based dynamic routing (LBDR) protocol and argued that localization is beneficial in dynamic underwater networks where nodes frequently change their positions. The network is divided into layers. A routing pipe is constructed within these layers. The movement of the nodes with water currents causes the nodes to move in and out of the routing pipe. The protocol achieves high throughput as packets are forwarded directionally in the pipe towards the water surface. However, the data load is high on the nodes in the pipe if the water currents do not frequently bring new nodes into the pipe.

#### 3.1.2. Protocols Addressing Energy Balancing

These protocols involve energy balancing and ensure that certain nodes within the network do not get overburdened due to frequent data forwarding. This is because the overburdened nodes deplete their battery power and die soon. The death of such nodes interrupts the reliable delivery of data to the surface of the water. Generally, nodes close to the surface of the water are overburdened as these nodes are heavily involved in data routing due to the closeness to the water surface. The description of these protocols follows below.

##### HH-VBF

In order to overcome the overburdening of the nodes in the routing pipe of VBF, a hop-by-hop VBF (HH-VBF) protocol is proposed [20] that defines the routing pipe for every forwarder node in contrast to the single major pipe in VBF. However, defining the routing pipe for every forwarder node increases the computational delay. In addition, nodes in the pipes are still preferred for selection as forwarders. This overburdens such nodes.

##### REBAR

A reliable and energy-balanced routing (REBAR) protocol establishes a varied radius cylindrical path from the source to the destination [21]. This path has a lower radius near the sink to involve fewer nodes. This avoids early death of such nodes and achieves energy balancing. The protocol assumes that every node has knowledge of its own position and the position of the final destination. A source node incorporates the information of the established path and its location in the packet and broadcasts it. Upon receiving the packet, a neighbor node calculates the difference between its own distance and the distance of the source from the water surface. If this difference is smaller than a certain limit, it forwards the packet and otherwise drops it. Redundant packets transmission is suppressed by using a history buffer. Furthermore, the protocol overcomes the void problem by making nodes near a void transmit the packets to all its neighbors that are independent of their positions and distances from the sink. Nodes’ movement with water currents is used as a positive parameter to select different nodes to further achieve energy balancing. Routing in the restricted cylindrical path achieves energy efficiency, but compromises the number of successful packet receptions at the sink, especially in sparse conditions. Furthermore, significant node movement may cause an extra amount of energy consumption in position calculation.

##### BEAR

The balanced energy adaptive routing (BEAR) protocol considers a hemispherical network and divides it into sectors of equal radii [22]. Nodes that reside closer to the sink are assigned more power because of greater data load on them than the farther nodes. In addition, the density of nodes is kept higher near the sink than the rest of the network. Every node in a sector selects a forwarder node in the sector above it until data packets reach the sink. The protocol balances energy consumption and, therefore, prolongs the network lifetime. However, the protocol suffers from high interference near the sink as more nodes are deployed near the sink and their packets’ transmission is not monitored by defining the packet holding time.

##### MDA-SL

A message dissemination approach for storage-limited (MDA-SL) UWSNs involving opportunistic routing is proposed in [23] to track underwater objects. The scheme evaluates the messages received by the nodes to forward or discard packets. Data packets are forwarded towards nodes with high mobility or residual energy. When the memory elements of nodes are full, all newer messages are discarded. In this way, the throughput is optimized. However, nodes with high mobility and residual energy die rapidly due to overloading, which creates energy holes in the network. In addition, packets are lost when the storage elements of nodes are full.

##### NGF

The new greedy forwarding (NGF) protocol divides the number of packets to transmit among the forwarding nodes if more than two forwarders are involved to forward packets [24]. The division involves the Chinese remainder theorem. This reduces the overall routing load per node and achieves energy balancing, which as a consequence, increases the network lifetime. However, involving many nodes in data forwarding increases interference.

#### 3.1.3. Protocols Mitigating Channel Conditions

These protocols take into account the properties of the channel such as the link state condition, interference, noise or any other parameter associated with the channel that hinders the delivery of the packets to the water surface. These protocols are described below.

##### DFR

Directional flooding-based routing (DFR) considers the link quality to route packets from a source to a destination [25]. Every node knows its own position and the position of the forwarder and the sink (final destination at the surface of the water). Every node also knows the link quality of its one-hop neighbors. The protocol forwards packets using flooding in a restricted zone formed by the angle among the source, forwarder and sink. It also ensures that there is at least one forwarder in the flooding zone to avoid packet loss. Under the worst link conditions, the flooding zone can be extended to include more forwarders and then select the link with the best quality to forward packets. Redundant packet transmission is the major issue with this protocol, which unnecessarily consumes the energy of nodes.

##### QoSDFR

The quality of service-aware directional flooding-based routing (QoSDFR) improves the DFR protocol in that the sink transmits feedback towards the nodes about the link quality [26]. This is contrary to the DFR where every node computes the link state condition. On the basis of the feedback from the sink, every sender node adjusts the number of relay nodes either along the full or partial routing paths. The feedback from the sink reduces energy consumption as the nodes do not have to check the link condition repeatedly. However, it brings a high end-to-end delay as nodes have to wait for the feedback from the sink.

##### LASR

The authors in [27] modify the dynamic source routing (DSR) to location-aware source routing (LASR) and include two more features. The first feature signifies that nodes are aware of their positions as the network topology changes with water currents. The second feature includes link quality to route packets along the paths with minimal noise and interference. However, the route information may not be updated in time due to high propagation delay in underwater communications. As a result, a forwarder is selected on the basis of old route information. Consequently, the best forwarder nodes may not be selected. For instance, a forwarder may change its position with water currents or its route information may change due to the unpredictable and dynamic nature of the underwater medium. At the same time, other nodes may not become aware of these changes due to high propagation delay in underwater communications.

##### SMIC

The sink mobility with incremental cooperative (SMIC) routing protocol considers the amplify and forward cooperative routing strategy to send packets from a source to a destination [28]. The relay is selected based on its residual energy, depth and quality of the link. A mobile sink gathers data packets from the destination nodes. The protocol shows improved performance in terms of packets received by the sink and network lifetime. However, the use of cooperative routing involves high energy consumption because a source node and a relay node both send the same data packets to a destination node (cooperative routing).

##### EGRCs

An energy-efficient grid routing based on 3D cubes (EGRCs) addresses the inherent challenging properties of the underwater medium [29]. The entire network is considered as a cube that is further divided into smaller cubes called clusters. A cluster head is selected based on its residual energy and location information. Every cluster head further selects a relay node based on residual energy, end-to-end delay and location. The protocol improves end-to-end delay, energy consumption and throughput. However, the death of a cluster head severely degrades system performance.

##### MMBR

Markov model-based routing (MMBR) probabilistically selects routes from the bottom to the top that are more stable, adaptable with respect to the varying data traffic and have fewer hops [30]. However, the movement of nodes with water currents changes the positions of the nodes, and therefore, relay nodes may not be accessed at their calculated locations.

##### EEIAR

An energy-efficient interference-aware routing (EEIAR) protocol is proposed in [31]. A source node selects a forwarder node that has the shortest path to the destination and the least number of neighbors. Shortest path selection reduces propagation delay. The selection of a forwarder node with the least number of neighbors minimizes interference during packet forwarding. However, nodes close to the water surface die rapidly due to constant selection of the shortest path; as these nodes provide the shortest paths.

#### 3.1.4. Protocols Addressing Energy Consumption

These protocols address the overall energy consumption in UWSNs. They reduce the overall energy consumption of the network for sustained operation of the sensor nodes. A description of these protocols is given below.

##### FBR

In focused beam routing (FBR) protocol [32], a source node sends a control packet to its one-hop neighbors to inform them about location information of itself and of the final destination. However, not all neighbor nodes respond to the packet. Only neighbors that lie within a cone formed by the angle between the source node and the ultimate destination respond to the packet. Every node calculates its position with respect to the straight line connecting the source node and the ultimate destination to determine whether or not it is located within the cone. A sender node uses many power levels to communicate with its neighbors. It starts with the lowest power level and increases it until it receives replies from the suitable neighbors. When the source node does not get any reply from neighbors even with the maximum power level, it increases the size of the cone to find suitable forwarder nodes. Since relay selection is accomplished within the limited zone, the protocol may not work efficiently in sparse conditions.

The routing path in FBR is a cone from sender towards the receiver while it is a pipe (cylinder) in VBF. Unlike the fixed size of the pipe, the cone size is adjustable in VBF to avoid the situation when a sender node has no neighbor node. In addition, VBF gradually increases the transmit power level from source to destination to avoid unnecessary power consumption.

##### MC

The protocol in [33] achieves the maximum coverage (MC) of the network in terms of data gathering from the sensor nodes using two mobile sinks. The mobility of the sinks in a circular fashion ensures the consumption of the energy of nodes in a balanced manner and also reduces packet loss. However, rather than moving to the targeted locations, where nodes have data to send, the sinks follow the predefined circular paths. This leads to dropping out of some packets and increases the delay, especially in sparse conditions.

The difference between MC and SBR-DLP is that the former uses two mobile sink nodes while the latter uses only one. This allows greater coverage of the network in the former than the later. In addition, the sink nodes in MC follow circular trajectories to cover the network, while the movement of the sink (destination) node is random in SBR-DLP. This eliminates the need for sensing a specific underwater zone.

##### BEEC

The balanced energy-efficient circular (BEEC) routing protocol [34] considers a circular network divided into ten circular regions called sectors. Two mobile sinks collect data from these sectors; one sink for five different sectors. The sinks move in a circular pattern to gather data from source nodes. This reduces and balances the energy consumption of the nodes and enhances the number of successful packet receptions by the sinks. However, the sinks do not prioritize the locations where nodes have data to send. They follow a fixed pattern of movements independent of the network conditions, which leads to packet loss and delay. Furthermore, the protocol performs poorly in sparse conditions when nodes are far apart from one another.

##### VBF, HH-VBF and FBR

The authors in [35] performed a comparative study of the VBF, HH-VBF and FBR protocols. They concluded that for the selected underwater scenario, when the transmission range of a sensor node equals the total depth of the network, VBF has the longest end-to-end delay and network lifetime. Conversely, the HH-VBF has the highest number of packet receptions at the sink, but has the shortest network lifetime.

##### MEES

The mobile energy-efficient square (MEES) routing protocol uses two mobile sinks at the longest possible distance from each other [36]. Their movements follow predefined linear paths to collect data from the nodes. The protocol improves throughput, energy consumption and network lifetime. However, rather than moving to the locations where nodes have data ready for transmission, the sinks follow predefined paths that cause packet loss and delay. In addition, nodes drop packets when the sinks are not within their communication range. Furthermore, the network performance is poor in sparse conditions.

##### LOTUS

The range-based low-overhead localization technique (LOTUS) relaxes the condition of using four sensor nodes as reference nodes to accomplish the localization of nodes [37]. It uses two reference nodes to localize nodes. This relaxation is helpful in identifying the position information of nodes in sparse conditions. However, with two reference nodes, the probability of error in position estimation of the nodes is higher than in four nodes.

##### GDflood

The geographical duplicate-reduction flooding (GDflood)-based routing protocol [38] considers the knowledge of the localization information of sensor nodes and combines it with network coding to overcome high energy consumption and duplicate packet transmissions. Energy consumption of the network is reduced by taking into account only suitable forwarders based on their position information. The network coding combines multiple packets into one or more single packets to reduce duplicate packets. However, the end-to-end delay is high due to the use of acknowledgment messages during data forwarding among the nodes.

##### DTMR

The authors in [39] argue that signaling overhead in UWSNs is costly as the sensor nodes have limited battery power. Furthermore, this adds to delay in the communications. Therefore, to reduce the energy consumption and delay, a source node sends the data directly to the destination node or through a mobile relay node. This is called direction transmission or mobile relay (DTMR) communications. The choice of sending data directly to the destination or through the mobile relay is depicted as a trade-off between the delay and the throughput of the network. However, direct transmission to the destination may not guarantee reliable delivery of data packets as the underwater medium is highly unpredictable and harsh.

##### FVBF

The authors in [40] argue that in selecting forwarder nodes in VBF, the position information of the sensor nodes alone is not enough to select the best forwarders. This is because the underwater environment is unpredictable and harsh and nodes change their positions and drain their battery power. As an improvement to VBF, a fuzzy logic-based vector-based forwarding (FVBF) protocol is proposed. The forwarder nodes are selected based on the valid distance, projection and the level of the battery power of the sensor nodes. The valid distance determines the extent to which a node is close to the sink node. The projection of a node determines its closeness to the routing pipe. The protocol achieves efficiency in energy consumption, throughput and end-to-end delay. However, nodes in the selected cylindrical vector die rapidly due to the heavy load of data, as they are frequently selected for data forwarding.

#### 3.1.5. Protocols Addressing Void Region

These protocols avoid the condition when a sender node does not find any neighbor node for data routing. The absence of a neighbor node of a sender node is either due to the placement of the sender node in a region where no neighbor exists or when all of its neighbor nodes die. The void condition severely affects the data delivery during routing, which as a consequence, leads to data loss. Protocols that take into account the void region are below.

##### AVN-AHH-VBF

The avoiding void node with adaptive hop-by-hop vector-based forwarding (AVN-AHH-VBF) protocol improves the VBF protocol in three ways [41]. Firstly, every node forwards a packet only if the next hop is not a void region. A void region is the one where only one neighbor exists for the source node. In this case, the source node drops the packet (rather than forwarding it to the void) to reduce energy consumption. Secondly, the depth of a relay node in the pipeline is also taken into account to receive packets from a source node and forward them to the sink. This ensures that packets reach the sink in less time. Thirdly, the holding time considers the number of hops a packet has traversed and the number of neighbors of the node holding the packet. This reduces end-to-end delay. However, the protocol reduces energy consumption at the expense of packet loss. It also suffers from the major problem with VBF: nodes in the pipe are overloaded by data traffic.

##### OVAR

The opportunistic void avoidance routing (OVAR) protocol uses opportunistic routing to address the void problem [42]. In OVAR, every node is capable of adjusting its number of neighbor nodes in the forwarder set to transmit data to the destination. The forwarder set is selected for a node on the basis of packet delivery probability and packet advancement. The protocol improves system performance in terms of throughput, energy consumption and end-to-end delay. However, it involves unbalanced energy consumption of nodes as nodes close to the sink are frequently selected for data forwarding.

##### GEDAR

The authors in [43] propose a geographic and opportunistic routing with depth adjustment-based topology control for communication recovery (GEDAR). The protocol forwards packets from the source to the destination in a greedy and opportunistic fashion. A source node selects the set of forwarding nodes based on packet advancement. The packet advancement is obtained by subtracting the distance between a neighbor node and the destination from the distance between the source node and destination. In the recovery mode, when a node finds itself in the void region (has no neighbors at all), it informs its two-hop neighbors. Based on the received location information of the two-hop neighbors, it decides the new depth location to avoid the void zone. The selection of the forwarder set can be done at the source, relay or receiver side. Furthermore, the coordination methods among the forwarder nodes are either timer based or control packet based. The latter, however, leads to high energy consumption as the exchange of control packets among the sensor nodes consumes energy. In addition, the opportunistic routing selects nodes close to the water surface, which leads to unbalanced energy consumption.

##### EHCAR

The energy hole and coverage avoidance routing (EHCAR) in [44] overcomes energy and coverage holes. When the residual energy of a node falls below a certain threshold, it broadcasts its status to the neighbor nodes. All the nodes receiving the broadcast message further forward the message. Other nodes then move to the position of the node with energy lower than the threshold. The movement of nodes is controlled in a manner that new holes are not created in response to covering the already created holes. However, a hole may change its position before a high residual energy node takes its position due to high propagation delay and communication among the nodes as to which node will move to the hole position. This leads to false position estimation of the node with residual energy below the threshold.

##### PAM

The position-aware mobility (PAM) of underwater autonomous vehicles (AUV’s) to avoid void zones is proposed in [45]. From the predefined positions of the gliders at the start of the sojourn tour, the direction of motion, current positions and distance from the neighbor gliders and the mobility patterns are defined and estimated, so no void zone is left. The transmit power of the gliders is also varied in the defined mobility patterns. However, the mobility model consumes too much energy of the gliders and causes high propagation delay when gliders are far apart.

##### FLMPC

The authors in [46] modified the layered multipath power control (LMPC) algorithm into the forward layered multipath power control-one (FLMPC-One) and forward layered multipath power control-two (FLMPC-Two) algorithms. The network is segmented into layers, and the source nodes are placed at the bottom layer. All the sensor nodes are randomly deployed in the network and initially are in sleeping mode. Upon receiving data packets, the nodes leave sleeping mode and become active. Unlike LMPC, a source node in FLMPC-One ensures that a forwarder node has neighbor nodes till two hops before selecting it as a forwarder node. FLMPC-Two ensures that a neighbor node has three hops. These strategies control the absence of forwarder nodes and, therefore, control the loss of data packets. They also avoid unnecessary forwarding of data packets, which leads to energy efficiency. However, checking the number of hops of the neighbor nodes increases the end-to-end delay.

##### VBVA

To address the void problem in three-dimensional mobile underwater sensor networks, the authors in [47] proposed a routing protocol called vector-based void avoidance (VBVA). This protocol has two features to cope with the void problem. Firstly, it uses a vector shift that directs the flow of data along the boundary of a void during routing. Secondly, when a concave void is along the way, a back-pressure strategy reverses the packets back along the paths. This leads to reliable delivery of data packets to the surface of the water. However, the energy consumption is not optimal due to the two selected features for void handling. This is because these features consume an extra amount of energy in selecting the routes and reversing the packets back along the paths with a concave void.

### 3.2. Localization-Free Routing Protocols

These protocols do not require the two- or three-dimensional position coordinate information of the sensor nodes. Instead, they use pressure sensors with the sensor nodes to measure the pressure of water on the sensor nodes. This water pressure is then used as a measure of the depth of the sensor nodes to construct and follow the routing paths. These protocols have the advantage of making the network easily scalable. This is because only the depth or pressure information is required, which can be obtained easily by the pressure sensor attached to the sensor node. This makes these protocols the best choice for underwater applications where scalability is desired such as underwater military operation, surveillance and underwater exploration. This is because these applications usually require exploring a wide area of the sea rather than a limited area. These protocols are described in the lines to follow. The categories of classification are the same for these protocols as the localization-based routing protocols. Each of these categories is described below. Table 3 shows a summary of these protocols.

#### 3.2.1. Protocols Addressing Node’s Mobility

##### DBR

Depth-based routing (DBR) is the pioneering protocol in the transformation from localization-based to localization-free routing in UWSNs [48]. This protocols uses the mobility of the sensor nodes to add scalability to the underwater networks. The water pressure (depth) of the sensor nodes is used as a forwarders’ selection parameter. With this parameter, the movements of the nodes with ocean currents do not require knowing the change in position of the sensor nodes. The DBR deploys five static sinks at the surface of the water and two source nodes at the bottom of the network. Source nodes sense the desired attribute and forward the data packets towards the sink in a flooding manner. Every node inserts its depth and ID information in the data packets to send. Upon receiving a data packet, every node holds it for a certain time, called the holding time. A forwarder node forwards a received packet if it comes from a higher depth node and otherwise discards it. The DBR has a better packet delivery ratio and end-to-end delay due to the selection of nodes with the lowest depth as relays. However, it suffers from redundant packets and high load on the nodes close to the sinks (low depth). Such nodes die soon and create energy holes in the network. These holes affect the system performance in the later stage of network operation.

##### DSRP

Akanksha and his colleagues investigated the effect of the mobility of nodes in the distributed delay-sensitive routing protocol (DSRP) [49]. The random Waypoint model is considered to address the mobility of nodes in the network. This model involves changes in position and velocity of the sensor nodes to move to a new destination. At each destination, nodes stop momentarily and then move to new positions. The proposed routing strategy involves the movement style of nodes, the expected traffic along the chosen paths and the localization of nodes. The speed with which nodes move is kept between the maximum and minimum values. The conditions of no mobility, low mobility and high mobility of nodes are compared. Based on simulation results, the proposed protocol shows that high mobility nodes have the greatest number of packets received at the final destination at the expense of the highest energy consumption and end-to-end delay.

#### 3.2.2. Protocols Addressing Energy Balancing

##### EEDBR

The energy-efficient depth-based routing (EEDBR) protocol considers the residual energy of nodes in addition to the depth to counteract the effect of the rapid death of low depth nodes in DBR [50]. When a sender node has to select a forwarder node, it selects a forwarder with the lowest depth and the highest residual energy among its one-hop neighbors. This leads to energy balancing and avoids energy holes in the network. However, the EEDBR does not guarantee the reliability of data at the final destination. This is because only a sender node decides the next forwarder in EEDBR, which may increase the probability of packet loss if the link quality is not good.

##### ODBR

The optimized depth-based routing (ODBR) protocol [51] assigns initial energy to nodes based on their vertical distances (depth) from the surface of the water. Nodes closer to the water surface are, therefore, assigned more energy than nodes farther from it. This results in balanced consumption of energy and prolongs the network lifetime. However, this protocol only works in shallow water zones. It does not perform well in networks that require deployment of sensor nodes at the bottom of the ocean because such nodes are assigned the least amount of energy.

##### EBECRP

The authors in [52] design an energy-efficient and balanced energy consumption cluster-based routing protocol (EBECRP) that divides the network into sectors. Each sector is assigned a cluster head to collect data from its neighbors and reduce multi-hoping. Two mobile sinks monitor the dense and sparse regions of the network based on the number of neighbor nodes. Nodes either send the data to the sinks or to the cluster heads. The sinks then collect the data from the cluster heads. The protocol prolongs the network lifetime by reducing energy consumption. However, the cluster heads are overloaded and die rapidly, which causes packet loss. Furthermore, the movement of cluster heads with water currents is a major issue that leads to packet loss.

##### Hydrocast

In the protocol proposed in [53], sensor nodes use water pressure in an opportunistic fashion to forward data packets to the surface sink. The selected set of forwarder nodes maximizes routing in a greedy manner and reduces energy balancing. A dead and recovery method is also proposed to ensure that packets reach the destination. The protocol achieves energy balancing, high packet reception at the sink and low end-to-end delay. However, in sparse conditions, the dead and recovery method does not perform well as nodes do not find enough neighbors. This compromises the performance of the proposed scheme. In addition, opportunistic routing prefers nodes close to the water surface as data forwarders. Such nodes die rapidly due to the high data load on them.

##### OMR

An optimal multimodal routing (OMR) protocol is proposed in [54] that combines acoustic and RF technologies during data forwarding. The selection of a technology is made to avoid bottlenecks and overburdening of relay nodes, as well as to avoid redundant data transmissions during packet forwarding. Every sensor node is assigned fair resources and technologies to participate fairly in data routing.

As shown in Table 1, long range communications have a very narrow bandwidth. That means less data can be sent in long-range communications. Therefore, the use of acoustic waves in OMR for long-range communication results in reduced throughput. The use of radio waves in OMR cannot be used for long-range communications since these waves are severely attenuated by water.

#### 3.2.3. Protocols Mitigating Channel Conditions

##### DEAC

The depth- and energy-aware cooperative (DEAC) routing protocol uses cooperative communications to avoid data corruption by the underwater medium [55]. The protocol adaptively selects a depth threshold for a source node based on its number of alive neighbors. Following this, a relay node is selected within the depth threshold based on its residual energy, number of neighbors and link state. The destination node is selected out of the depth threshold. The protocol performs well in terms of packets received at the sink. However, it is energy inefficient due to cooperative routing.

##### DBR-NC

Depth-based routing network coding (DBR-NC) improves the energy cost of packets transmission, delay and packets’ reliability in the original DBR scheme. The network coding takes into account the adverse channel effects to ensure reliability. However, the protocol assumes an ideal MAC layer compared to the realistic MAC layer in DBR [56].

##### QERP

The authors in [57] proposed a quality-of-service (QoS)-aware evolutionary cluster-based routing protocol (QERP) to mitigate the adverse channel effects in underwater communications. The protocol uses the genetic algorithm, divides the network into clusters and specifies the cluster heads and the routing paths from the routing tables that nodes share with one another. Packet delivery ratio, end-to-end delay and energy consumption are improved. However, the load on the cluster heads makes them depleted of energy at the early stage, which creates energy holes in the network and degrades system performance.

##### RIAR

Majid and his colleagues propose a reliable and interference-aware routing (RIAR) protocol to mitigate the unwanted link effects in routing data from the source to the destination [58]. A source node selects a relay node on the basis of its number of hops from the surface sink, neighbors and the greatest distance from the source to the relay node. These parameters are combined to propose a cost function. A relay node with the highest value of the cost function is the suitable candidate for data forwarding. The protocol performs well in sparse networks in terms of throughput, energy consumption and end-to-end delay. However, it causes early the death of nodes close to the water surface, which reduces the number of successful packets received at the sink.

##### EEIRA

To address interference during packet forwarding, an energy-efficient interference- and route-aware (EEIRA) protocol is proposed in [59]. The protocol selects forwarder nodes based on the lowest depth and the least number of neighbors of the forwarder nodes. These parameters ensure efficient utilization of energy and reduced interference. However, the protocol performs poorly in sparse conditions where the selection of forwarder nodes with the least number of neighbors increases the probability of not finding a forwarder node by a sensor node.

##### EECOR

The authors in [60] use cooperative opportunistic routing to mitigate adverse channel conditions during packet forwarding. The cooperative routing ensures that the destination receives copies of the same data packets from the source and the relay for reliable reception of the packets. The opportunistic routing ensures that a certain set of the best forwarder nodes is chosen for data forwarding rather than selecting a single forwarder node. The forwarder nodes are selected based on fuzzy logic. The protocol shows promising performance in terms of packet delivery, energy consumption and end-to-end delay. However, constant checking and selection of the forwarder set add extra delay.

##### RRSS

A routing protocol based on received signal strength (RRSS) is proposed in [61]. A source node establishes a vector from itself to the sink node. The distance of the source node from the sink node is used as a measure of the intensity of the beacon signal. The beacon signal is broadcast by the sink node to communicate with the sensor nodes. A source node decides the selection of a forwarder candidate inside the vector based on the intensity of the hello packet and the intensity of the beacon signal at the forwarder. Nodes periodically broadcast hello packets to share vital information with one another. Forwarder nodes that are in the void regions are avoided to forward data packets. The protocol achieves improved energy efficiency and end-to-end delay. However, nodes inside the vector die rapidly due to data overloading.

#### 3.2.4. Protocols Addressing Energy Consumption

##### DRADS

The depth- and reliability-aware delay sensitive (DRADS) protocol modifies the concept of opportunistic routing [62]. It considers the depth information in addition to the link state information when a forwarder sends packets to the sink. This reduces energy consumption and increases the throughput. However, the loads on low depth nodes increase due to opportunistic routing, and they die soon, deteriorating the system performance.

##### DBR-MAC

In the DBR-MAC protocol [63], low depth nodes that suffer from high data load are prioritized to access the channel. The angle, depth and overhead of the neighbor nodes are considered for the low depth nodes to make them access the channel with greater preference than the rest of the nodes. This leads to improvement in energy consumption, throughput and end-to-end delay. However, prioritizing the low depth nodes overburdens them, and they die in a rapid fashion. This creates energy holes in the network, which leads to packet loss in the subsequent operation time of the network.

##### E-CARP

The authors in [64] improve the CARP protocol and make it localization free and more intelligent in selecting relay nodes. Packets are forwarded in a greedy hop-by-hop manner, and relay nodes are selected when the network conditions are steady. Energy consumption and network lifetime are improved in E-CARP at the expense of high end-to-end delay due to waiting for steady conditions.

##### SOR

The work in [65] designs a self-organizing (SOR) protocol where every node forwards the data packets of the nodes above it towards the gateway in a radial network. A hello packet is broadcast by the gateway to its one-hop neighbors. Once the neighbors respond, the gateway sends a route request to the neighbors, which process it until the last node sends an acknowledgment to the gateway. This information is used by the gateway to construct strings that forward the data packets. This process reduces the energy consumption by avoiding unnecessary forwarding of the data. It also shortens the end-to-end delay and increases the throughput. However, the protocol suffers from void problems when a node in the string dies, which results in packet loss. Furthermore, the mobility of nodes does not make it easy to form strings of nodes and forward the packets in a smooth manner.

##### DVRP

The diagonal and vertical routing protocol (DVRP) addressed in [66] reduces the number of forwarders among the source, relay and destination. Relay selection is accomplished by the adjustable angle from the source to the destination. A reduction in the number of forwarder nodes enhances the energy efficiency. The selection of the adjustable angle ensures the selection of routing paths with low end-to-end delay. In sparse conditions, the angle is increased to include one or more forwarder nodes. The protocol achieves a better packet delivery ratio than the counterpart scheme. However, the energy consumption of the protocol degrades when the number of sinks increases in the network.

##### DUCS

The distributed underwater clustering scheme (DUCS) [67] divides an underwater network into clusters. Every cluster has one major node called a cluster head that receives information from the rest of the one-hop neighbors in the same cluster. It then processes the information to remove redundant data and efficiently transmit the desired data to the sink using other cluster heads. A cluster head transfers the information of a node in one cluster to a node in another cluster. It achieves energy efficiency by reducing the number of control messages among sensor nodes through the cluster head. It also leads to a high packet delivery ratio. However, the movement of nodes with water currents seriously degrades the performance of the system. In addition, the cluster head is overloaded, and its energy is depleted rapidly, which creates void holes in the network and degrades its performance.

##### UMDR

The authors in [68] used the concept of space reuse and designed a routing protocol for ad hoc UWSNs. In space reuse, the coverage area is divided into different sectors, and nodes in each sector communicate with one another using directional antennas. This controls the redundant packet transmission, which in turn, controls energy consumption and interference. Furthermore, data packets are forwarded towards the desired destination directly without broadcasting, which reduces end-to-end delay. However, directional antennas require next-hop information and the antenna information of the next hop during every transmission. This introduces computational complexity to the routing process.

#### 3.2.5. Protocols Avoiding the Void Region

##### EBPR

Energy balanced pressure routing (EBPR) uses the feedback from sensor nodes for the beacon signals to avoid void zones [69]. It does not take into account the velocity of nodes to avoid void zones. In addition, residual energy is considered as a routing metric in selecting a forwarder node. Furthermore, the proposed protocol uses the redundant route information to help nodes that do not have this information to update route information. The EBPR increases the lifetime of the network. However, it suffers from poor performance in sparse networks when sending beacon signals is less effective.

##### CARP

Basagni and his co-workers proposed a channel-aware routing protocol (CARP) [70] that keeps in view the successful packet transmission history of nodes to deliver packets from the source to the destination. It combines hop count with a power control strategy to successfully deliver packets and avoid void and shadow zones. However, the end-to-end delay is larger in dense networks, where constant checking of the packet transmission history introduces delay.

##### LF-IEHM

A localization-free interference and energy holeminimization (LF-IEHM) routing protocol is proposed in [71]. It uses a variable transmission range of the sensor nodes to avoid the condition when a sensor node has no neighbor for data forwarding. In addition, a unique packet holding time is defined for every sensor node to reduce the probability of two or more nodes forwarding the packets at the same time. The protocol achieves high throughput at the cost of high energy consumption.

## 4. Localization-Based vs. Localization-Free Routing Protocols

Localization-based routing protocols use the two- or three-dimensional position coordinates information of the sensor nodes to construct the routing paths. This information is helpful in constructing the optimal routing paths from the bottom to the surface of the water, which may improve the performance parameters such as propagation delay and other parameters. However, the calculation of the position information of the sensor nodes is challenging as the sensor nodes move with ocean currents. Energy is also consumed in knowing and calculating the position information of the sensor nodes. These challenges compromise the performance of the localization-based routing protocols. These protocols are used in underwater object tracking where knowing the exact position of a target is necessary or any application that requires preciseness in the position calculation of the sensor nodes or the tracked objects.

Localization-free routing protocols do not require the two- or three-dimensional position coordinates information of the sensor nodes. Instead, these protocols make use of pressure sensors as a measure of the pressure of water on the sensor nodes, which in turn, measures the depth of the sensor nodes. The depth of the sensor nodes is then used to construct the routing paths. This strategy saves the amount of energy and reduces the propagation delay in the calculation of the position of the sensor nodes. These protocols are used when scalability of the network is desired, such as in underwater military operations or when general-purpose monitoring of the sea is the objective.

## 5. Conclusions

A survey of the current and state-of-the-art routing protocols for UWSNs is presented. Challenges associated with the underwater channel are characterized. The routing protocols are classified into two groups: localization-based and localization-free protocols. The former requires two- or three-dimensional position coordinates information of the sensor nodes for establishing the routing paths. The latter requires only the depth (or pressure) information. Each category of the protocols is further subdivided according to the problem(s) it addresses or the major parameter(s) it considers for routing. They include node’s mobility, energy balancing, channel properties, energy consumption and void zone. Every protocol is described in terms of its routing strategy, merit(s) and demerit(s). Such a description of the protocols is useful in a number of ways for researchers, as compared to the existing surveys. The routing strategies help in understanding the routing operation of the protocols. The demerits lead to the design of new protocols that are more efficient and effective. The merits help in the selection of the right protocol for the right underwater application. Open research challenges and directions that need to be addressed in the future are specified.

## 6. Open Challenges and Future Research Directions

In this section, open challenges and future directions are presented for UWSNs.

- The channel models for noise and attenuation calculation in UWSNs are empirical. This area is still open for the analysis and investigation of new models developed in an analytical or computational manner. In addition, experimental models can also be developed to reflect the characteristics of the underwater medium.
- Sensor nodes move with water currents by which they change their positions constantly. As a result, their position information also changes. This makes the localization of the sensor nodes a challenging task. The movement of nodes also requires constant updates about the new positions. This inherently adds delay and energy consumption as nodes have to exchange such information. Future research needs to address these issues.
- The routing protocols for UWSNs make use of the network layer to deliver data packets from the bottom to the surface of water. All the routing protocols described in this survey consider only the network layer. To optimize network performance, the network layer can be combined with the MAC layer to reduce the waiting time of the packets in the queues of the sensor nodes. This will also reduce the interference during packet transmission and, consequently, the energy consumption. This is because interference leads to packet loss, so the lost packets are retransmitted, which consumes additional energy.
- Since the underwater medium is highly unpredictable and challenging, protocols focusing on the reliability of data transmission from the bottom to the surface of the water are worth investigation in future research.
- In all the existing routing protocols, nodes close to the water surface are frequently selected as forwarders, since these nodes are close to the surface sink. This leads to high data load on these nodes. As a result, such nodes drain their batteries rapidly and die. The death of such nodes disconnects the data traffic from the bottom to the surface of the water, which as a consequence, threatens the reliable operation of the network. Future research needs to cope with the early death of the sensor nodes.
- The variation of the acoustic speed with depth, salinity and temperature of the water causes the acoustic waves to travel on curved paths. This creates regions where the sensor nodes are not able to communicate with other nodes in their vicinity. This is a challenging issue for the design of future routing protocols.

## Figures and Tables

**Table 1:** Convergence bandwidth relationship in UWSNs.

Convergence	Range (km)	Bandwidth (kHz)
Very long	100	Less than 1
Long	10–100	2–5
Medium	1–10	Almost 10
Short	0.1–1	20–50
Very short	Less than 0.1	Greater than 100

**Table 2:** Localization-based routing protocols.

Protocol	Routing Strategy	Problem Addressed	Merits	Demerits	Year
VBF	Selects forwarder nodes within a pipe from the source to the destination	Nodes’ mobility	Energy efficiency, scalability	Nodes in the pipe die quickly due to high data load, which creates energy holes (dead nodes)	2006
SBR-DLP	Makes the destination nodes mobile and divides the transmission range of a source node in such a manner that nodes close to the mobile destination communicate with the destination directly rather than communicating with the source node	Nodes’ mobility	High throughput as mobile destination nodes collect data packets	High delay as nodes have to wait for the mobile destination to collect packets from them because the destination nodes do not prioritize nodes that have packets ready for transmission	2009
NEFP	Combines the forwarding probability of a packet with packet holding time in a routing zone formed by the angle among the source, forwarder and destination	Nodes’ movement	Energy efficiency	Compromised performance in sparse conditions when a source node cannot find neighbor nodes in the restricted forwarding zone	2016
TC-VBF	Modifies the VBF protocol to select forwarders in response to nodes’ density	Nodes’ mobility	Energy efficiency	Low throughput when the number of nodes is small	2017
LBDR	The network is divided into layers, and nodes forward data packets from the bottom to top within a routing pipe in these layers	Nodes’ mobility for localization of forwarder nodes in a routing pipe	High throughput	High load on the nodes within the routing pipe if new nodes do not move into the pipe	2016
HH-VBF	Rather than defining a single routing pipe as in VBF, a separate pipe is defined for every forwarder node	Reduction of data load on the nodes in the single routing pipe of VBF	Minimization of energy hole formation in VBF by controlling data load on the nodes	High computational delay due to defining the routing pipe for every node, compromised throughput when nodes are far apart and the routing pipe cannot be made	2008
REBAR	Creates an adaptive cylindrical path from the source to the destination with a varied radius to choose forwarders	Energy balancing and early death of nodes close to the water surface	Energy efficiency and void hole control	Error in position calculation of nodes when they move	2007
BEAR	High energy nodes with greater density closer to the sink are selected	Energy balancing	Long network lifetime, balanced energy consumption	High interference near the sink as more nodes are deployed near the sink and their packet transmission is not controlled by the packet holding time	2016
MDA-SL	Messages forwarded to high mobility or residual energy nodes are evaluated to decide forwarders	Energy balancing by selecting different high energy nodes	High throughput	Unbalanced energy consumption causes energy holes as nodes with high mobility or residual energy are frequently selected for data forwarding and die soon	2016
NGF	Divides the packets between two or more nodes based on the Chinese remainder theorem	Load per node	Energy balancing	High interference as the number of nodes involved in the routing process increases	2016
DFR	Defines a restricted forwarding zone formed by the angle among source, relay and destination nodes and forwards packets in the flooding manner	Severe channel conditions	Mitigation of adverse channel conditions, high throughput	High energy consumption due to redundant packet transmission caused by flooding	2007
QoSDFR	Unlike DFR, every node does not compute the link quality. Instead, the sink node sends feedback to all the nodes about the channel conditions. Every node then selects its forwarder nodes accordingly	Energy consumption in DFR, estimation of dynamical channel conditions due to regular feedback from the sink to the nodes	High throughput due to estimation of changing channel conditions and routing accordingly, reduced energy consumption as every node does not compute the channel like in DFR	High end-to-end delay due to the nodes regularly waiting for the feedback from the sink	2014
LASR	Packets are forwarded along the routes with minimal noise and interference	Link state conditions	High throughput and balanced energy consumption	Latency in updating routes information leads to false forwarder’s positions estimation	2006
SMIC	Uses the incremental amplify and forward cooperative routing method	Adverse channel conditions	High throughput	High energy consumption due to cooperative routing where a source node and a relay node both send the same packets to the destination node	2016
EGRCs	A 3D cube network is sub-divided into cubic clusters with a cluster head selected in each cluster based on residual energy and position while relay nodes are selected based on residual energy, position and end-to-end delay	Channel properties	Energy efficiency, low end-to-end delay, high throughput	Degraded performance when a cluster head dies	2016
MMBR	Routes that are stable and adaptable with data traffic and have fewer hops from the source to the destination are selected	Noise and interference	Reliability	Nodes’ movement introduces error in position estimation of nodes	2016
EEIAR	A source node chooses a forwarder node with the least number of neighbors and the shortest path to the destination	Interference over channel during routing	energy efficiency, low end-to-end delay	Fast death of nodes close to the water surface as they provide the shortest paths	2017
FBR	Selects relays within a cone formed from the source to the destination	Energy consumption	Energy efficiency and low end-to-end delay	Low throughput in sparse conditions (when the network density is low and nodes are far apart)	2008
MC	Two sinks moving in a circular fashion get data from source nodes	Energy consumption	High throughput and balanced energy consumptions as the mobile sink collect data from the nodes	High delay as nodes have to wait for the mobile sinks to reach their range because the sinks do not prioritize nodes ready to send data	2016
BEEC	Two mobile sinks collect data from source nodes in a circular network	Energy consumption	Balanced and low energy consumption, high throughput	Sinks do not first move to locations based on priority where nodes have data to send, which causes packet loss and poor performance in sparse conditions	2016
VBF, HH-VBF and FBR	Perform a relative comparison of VBF, HH-VBF and FBR	Throughput and energy consumption	Longest network lifetime in VBF and highest throughput in HH-VBF	Longest end-to-end delay in VBF and shortest network lifetime in HH-VBF	2017
MEES	Two mobile sinks located at the farthest distance move in predefined linear paths to collect data from nodes	Energy consumption	Energy efficiency, energy balancing	The sinks do not move based on priority to locations where nodes have data to send, nodes drop packets when the sinks are not in their communication range	2017
LOTUS	Uses two instead of four reference nodes to position sensor nodes	Energy consumption	Energy efficiency and low end-to-end delay	Greater probability of error in position estimation of nodes	2016
GDflood	Uses localization of sensor nodes and network coding to combine more packets into one or more output packets	Energy consumption and duplicate packet transmission	Energy efficiency and high throughput	High end-to-end delay as nodes exchange acknowledgment messages during data forwarding	2016
DTMR	Uses direct transmission from the source to the destination or through a mobile relay	Energy consumption due to signaling overhead	High throughput and low delay	No reliability of data in the case of direct transmission	2018
FVBF	Nodes in a pipe from the source to the water surface are selected based on closeness to the sink, closeness to the shortest path from the source to the sink and the battery level	Energy consumption	High throughput, energy and delay efficiency	Nodes in the pipe overload and die rapidly	2018
AVN-AHH-VBF	Improves VBF by considering the void region, depth and the altering holding time of VBF	Void region, energy consumption	Energy efficiency	High packet loss to save energy, nodes in the pipe overloaded with data as in VBF	2016
OVAR	Uses opportunistic routing and selects relay nodes based on packet delivery probability and packet advancement	Void zone	Energy efficiency, high throughput, low end-to-end delay	Unbalanced energy consumption	2016
GEDAR	Uses opportunistic routing and selects forwarder nodes based on packet advancement	Energy consumption, void zone	Energy efficiency	Unbalanced energy consumption	2016
EHCAR	A node with energy below a certain threshold informs other nodes to replace it and avoid holes	Void zone	Energy efficiency, high throughput	False position estimation of the void node due to high propagation delay, movements of the nodes with water currents and communication among the nodes in choosing the suitable node to replace the void	2016
PAM	Autonomous underwater vehicles have predefined paths with known current distance and position from the neighbor vehicles to avoid the void zone	Void zone	High throughput	High energy consumption and delay when gliders are far apart	2016
FLMPC	A source node checks that a forwarder has two or three hops before sending packets to it to avoid packets loss due to the absence of neighbor nodes of the forwarder	Void region	High throughput as nodes having no forwarder in the next two or three hops are not selected; this also utilizes the energy efficiently	High end-to-end delay due to constant checking of the two and three hop information of the neighbor nodes	2018
VBVA	Void zone	A vector shift strategy routes the packets along the void’s boundary while a back-pressure mechanism reverse them when a concave void exists	High packets delivery	High energy consumption and delay due to packets reversing and defining the routes along the boundary of the void	2009

**Table 3:** Localization-free routing protocols.

Protocol	Routing Strategy	Problem Addressed	Merits	Demerits	Year
DBR	Uses the lowest depth sensor nodes from bottom to top to forward packets in a flooding manner	Nodes’s mobility to add scalability to the underwater networks	High throughput and relaxes the requirement of full dimensional position information of the sensor nodes	High energy consumption due to redundant packet transmission caused by flooding and early death of low depth nodes due to frequent selection as forwarder nodes	2008
DSRP	Nodes’ position and velocity changes are used to deliver packets to the surface sink	Node’s mobility	High throughput	High energy consumption and end-to-end delay	2016
EEDBR	Addresses the death of low depth nodes in DBR. A sender node decides the forwarder based on depth and residual energy	Energy balancing and death of low depth nodes	Balanced energy consumption and death of low depth nodes	Low reliability of packets delivery because a sender node of the data packets selects the forwarder nodes and sends a single copy of the data packets, which does not guarantee reliability when the channel conditions are unfavorable	2012
ODBR	Assigns more energy to nodes closer to the water surface	Energy balancing, early death of nodes closer to the water surface	Long network lifetime, balanced energy consumption	Does not work in deep water zones where bottom nodes should have enough energy to sense attributes	2016
EBECRP	Uses clusters and two mobile sinks to collect data from nodes	Energy balancing	Energy efficiency and balancing	Death or movements of cluster heads result in packet loss	2016
Hydrocast	Pressure-based opportunistic routing with the dead and recovery method to ensure packets are sent to the destination	Energy balancing	Energy efficiency	Compromised performance in sparse conditions, high data load of low depth nodes due to opportunistic routing	2016
OMR	Uses RF and acoustic waves in combination with fair resources for all nodes to avoid redundant packet transmission, overburdening of the relay nodes and bottlenecks	Energy balancing	High throughput, low end-to-end delay, energy efficiency	Cannot be used for long-range underwater communications due to fading of RF waves in water	2018
DEAC	Uses cooperative communications to avoid data corruption by channel	Adverse channel effects	Reliability and high throughput	High energy consumption due to cooperative routing	2016
DBR-NC	Combines network coding with DBR	Adverse channel conditions	Energy efficiency, high throughput and low end-to-end delay	Idealized and too simplified MAC layer	2016
QERP	Uses the genetic algorithm to divide the network into clusters and cluster heads for data forwarding	Severe channel conditions	High packet delivery ratio, energy efficiency and low packet latency	Cluster heads are overloaded and die early, which causes energy holes	2017
RIAR	Selects a forwarder based on its distance from the surface sink, source node and number of neighbors	Adverse channel effects	Reliability, energy efficiency	Early death of sensor nodes close to the water surface	2016
EEIRA	Selects forwarder nodes based on the lowest depth and the least number of neighbors	Channel properties (interference)	Energy efficiency	Poor performance when neighbor nodes are not available to forwarder nodes in low density networks	2016
EECOR	Uses cooperative opportunistic routing to forward packets to the final destination	Adverse channel conditions	Energy efficiency, high throughput	Communication among nodes in forwarder set adds delay	2017
RRSS	A sender node uses its distance from the surface sink and establishes a vector. Inside the vector, it chooses a forwarder node based on the received signal strength	Energy consumption	Energy efficiency	High data load on the nodes inside the vector	2017
DRADS	Considers the depth of the forwarders in addition to the links’ state	Energy consumption	High throughput, low end-to-end delay and energy efficiency	Early death of low depth nodes due to opportunistic routing	2016
DBR-MAC	Prioritizes forwarder nodes closer to the sink based on depth, angle and overhead	Energy consumption by preferring low depth nodes to participate in data routing	Energy efficiency, high throughput	Unbalanced energy consumption as nodes close to the water surface are selected frequently, drain their battery power and die soon	2016
E-CARP	Hop-by-hop forwarder selection in a greedy manner is accomplished when the network conditions are steady	Energy consumption	Energy efficiency	High end-to-end delay due to waiting for the channel conditions to become steady before selecting the relay nodes	2016
SOR	In the radial network, every node forwards packets of the nodes above them towards the sink using straight paths called strings	Energy consumption	Energy efficiency, low end-to-end delay	Data loss when a node in a string dies	2016
DVRP	Adaptively selects the flooding zone by the angle from a source node to the sink node through a relay node	Energy consumption	Energy and end-to-end delay efficiency, high throughput	Compromised energy efficiency when the number of sinks increases	2013
DUCS	Divides the network into clusters, and each cluster has a cluster head that forwards packets of other nodes to the sink	High energy consumption due to the exchange of messages among nodes	High packet delivery, energy efficiency	Nodes’ mobility and death of cluster nodes severely degrade system performance	2007
UMDR	The network is divided into sectors, and nodes in each sector communicate using directional antennas to avoid the broadcast nature of the routing	Energy consumption	High throughput, energy efficiency, low end-to-end delay	Frequent computation of the next hop and antenna information makes the routing process complicated	2018
EBPR	Feedback from the sensor nodes based on beacon signals and their residual energy is used to route data	Void zone	Energy efficiency	Compromised performance in sparse conditions where beacon signals do not work effectively	2016
CARP	Uses power control and successful packet transmission history of nodes	Void and shadow zones	High throughput, energy efficiency and low end-to-end delay	High end-to-end delay in dense conditions due to constantly checking of the successful packet transmission history	2015
LF-IEHM	Combines variable transmission range of nodes with packet holding time to avoid energy holes and interference	Void zone (combined with interference)	High throughput	High energy consumption	2018
